# Diagnostic markers of urothelial cancer based on DNA methylation analysis

**DOI:** 10.1186/1471-2407-13-275

**Published:** 2013-06-04

**Authors:** Yoshitomo Chihara, Yae Kanai, Hiroyuki Fujimoto, Kokichi Sugano, Kiyotaka Kawashima, Gangning Liang, Peter A Jones, Kiyohide Fujimoto, Hiroki Kuniyasu, Yoshihiko Hirao

**Affiliations:** 1 Department of Molecular Pathology, Nara Medical University, 840, Shijyo-cho, Kashihara, Japan; 2 Department of Urology, Nara Medical University, 840, Shijyo-cho, Kashihara, Japan; 3 Division of Molecular Pathology, National Cancer Center Research Institute, 5-1-1, Tsukiji Chuo-ku, Tokyo, Japan; 4 Department of Urology, National Cancer Center Hospital, 5-1-1, Tsukiji, Chuo-ku, Tokyo, Japan; 5 Oncogene Research Unit/Cancer Prevention Unit, Tochigi Cancer Center Research Institute, 4-9-13, Yonan, Utsunomiya, Japan; 6 Department of Urology, Tochigi Cancer Center Hospital, 4-9-13, Yonan, Utsunomiya, Japan; 7 Department of Urology, Norris Comprehensive Cancer Center, University of Southern California, 1441 Eastlake Ave, Los Angeles, CA, 90033, USA

**Keywords:** Urothelial cancer, DNA methylation, Pyrosequencing, ROC, Piagnostic accuracy

## Abstract

**Background:**

Early detection and risk assessment are crucial for treating urothelial cancer (UC), which is characterized by a high recurrence rate, and necessitates frequent and invasive monitoring. We aimed to establish diagnostic markers for UC based on DNA methylation.

**Methods:**

In this multi-center study, three independent sample sets were prepared. First, DNA methylation levels at CpG loci were measured in the training sets (tumor samples from 91 UC patients, corresponding normal-appearing tissue from these patients, and 12 normal tissues from age-matched bladder cancer-free patients) using the Illumina Golden Gate methylation assay to identify differentially methylated loci. Next, these methylated loci were validated by quantitative DNA methylation by pyrosequencing, using another cohort of tissue samples (Tissue validation set). Lastly, methylation of these markers was analyzed in the independent urine samples (Urine validation set). ROC analysis was performed to evaluate the diagnostic accuracy of these 12 selected markers.

**Results:**

Of the 1303 CpG sites, 158 were hyper ethylated and 356 were hypo ethylated in tumor tissues compared to normal tissues. In the panel analysis, 12 loci showed remarkable alterations between tumor and normal samples, with 94.3% sensitivity and 97.8% specificity. Similarly, corresponding normal tissue could be distinguished from normal tissues with 76.0% sensitivity and 100% specificity. Furthermore, the diagnostic accuracy for UC of these markers determined in urine samples was high, with 100% sensitivity and 100% specificity.

**Conclusion:**

Based on these preliminary findings, diagnostic markers based on differential DNA methylation at specific loci can be useful for non-invasive and reliable detection of UC and epigenetic field defect.

## Background

According to the American Cancer Society estimates for 2013, bladder cancer will account for 72,570 newly diagnosed cases and 15,210 deaths [1]. Bladder cancers can be classified into two groups based on histopathology and clinical behavior: non-muscle-invasive urothelial cancer (NMIUC: pTa-pT1) and muscle-invasive urothelial cancer (MIUC: pT2-pT4). NMIUCs represent approximately 80% of newly diagnosed bladder cancer cases and are treated by transurethral resection (TUR). However, 70% of the treated cases recur, and of these 15% progress to invasive cancers [2]. Consequently, the follow-up for NMIUC includes lifelong cystoscopy monitoring every few months. MIUC usually requires radical cystectomy and has a poor prognosis [3]. Although cystoscopy and cytology are the gold standard for diagnosing bladder cancer, cystoscopy is an invasive procedure and cytology has poor sensitivity for detecting low grade tumors [4]. It is therefore crucial to develop reliable and non-invasive early diagnostic markers to improve strategies for management of bladder cancer patients.

Genetic and epigenetic factors are known to contribute to the occurrence of bladder cancer [2]. Hence, several DNA-based urinary markers have been evaluated with the aim of reducing the need for cystoscopy and improving the accuracy of tumor detection. However, none have been proven to be sufficiently reliable in detecting the entire spectrum of bladder cancers in the clinic [5].

Among the recently developed diagnostic markers for bladder cancers, those based on aberrant DNA methylation appear to be highly promising. Recent findings have indicated that epigenetic silencing associated with various cancers may involve DNA methylation extending over a large chromosomal region, often described as genome-overall hypomethylation or regional hypermethylation [6,7]. Diagnostic indicators based on DNA methylation have potential advantages over other genetic markers because DNA methylation occurs widely in cancer cells and consistently affects the same promoter regions. Therefore, a minimal analysis using a few loci is sufficient for diagnosis [8]. Furthermore, there is accumulating evidence that aberrant DNA methylation occurs frequently and early in human carcinogenesis [9,10]. Several studies on bladder cancer have indicated that tumor-specific DNA methylation markers have higher sensitivity and specificity than the parameters used in cytological urine analysis [11,12]. However, when used in highly sensitive, quantitative analytical techniques for measuring DNA methylation in urine samples, these markers tend to lose their both sensitivity and specificity for cancerous cells [13-15]. One of the reasons for this could be that aberrant DNA methylation occurs in non-cancerous tissue also due to aging, smoking and environmental factors [6]. Secondly, both cancer cells and normal transitional cells shed in the urine may have altered DNA methylation because of concomitant conditions, especially chronic inflammation and/or persistent infection [16], or the urine samples may be contaminated with other types of cells. Moreover, most studies analyzed a region within a CpG island (CGI) that may be altered in its methylation status, but may not affect gene expression in non-cancerous regions. Quantitative DNA methylation methods are advantageous as these can detect pre-malignant epigenetic field defects that cannot be revealed by histological examinations.

We previously reported aberrant DNA methylation occurring in urothelial cancer (UC) through a genome-wide approach [17]. The aim of the present study was to select and validate markers based on UC-specific regional aberrant DNA methylation. The association of UC with aberrant DNA methylation in selected loci was analyzed statistically by comparison of malignant and normal urothelial tissues. Lastly, we assessed the clinical relevance of the identified markers for detecting UC using urine samples.

## Methods

### Sample collection and preparation

Tissue samples were collected at 4 participating centers following protocols approved by an institutional review board: (1) University of Southern California, Norris Comprehensive Cancer Center, and 3 Japanese institutions, (2) Nara Medical University, Nara, (3) National Cancer Center Hospital, Tokyo, and (4) Tochigi Cancer Center Hospital, Tochigi. Informed consent was obtained from all participants at the respective institutions, and this study was approved by Nara Medical University Medical Ethics Committee as the project name “Epigenetic profiling and diagnostic markers of urogenital cancer based on DNA methylation analysis” from October 5, 2010.

Tissue samples of tumor and corresponding normal-appearing tissue adjacent to the tumor were obtained from UC patients during the surgical procedure (TUR or radical cystectomy). Corresponding normal-appearing tissue were judged macroscopically or endoscopically and dissected. A half of tissues were taken pathological examination, if the tissue included cancer, the section was excluded for the analyses. Control tissue samples of normal urothelia were obtained from patients without UC. Tumors were staged according to the UICC 1987 TNM Classification system [18]. All collected tissues were frozen and stored at −80°C until use for DNA extraction.

Urine samples were collected from UC patients before surgery and from healthy volunteers by spontaneous urination. Voided urine samples (50 mL) were centrifuged at 2000 × g for 10 min, and the pelleted urine sediment was rinsed twice with phosphate-buffered saline (PBS) and stored until use for DNA extraction.

DNA was extracted using conventional extraction methods [19]. DNA (2 μg) was treated with sodium bisulfite using Epitect Bisulfite Kit (Qiagen) according to the manufacturer’s protocol and resuspended in 40 μL of distilled water for subsequent use.

**Figure 1:**
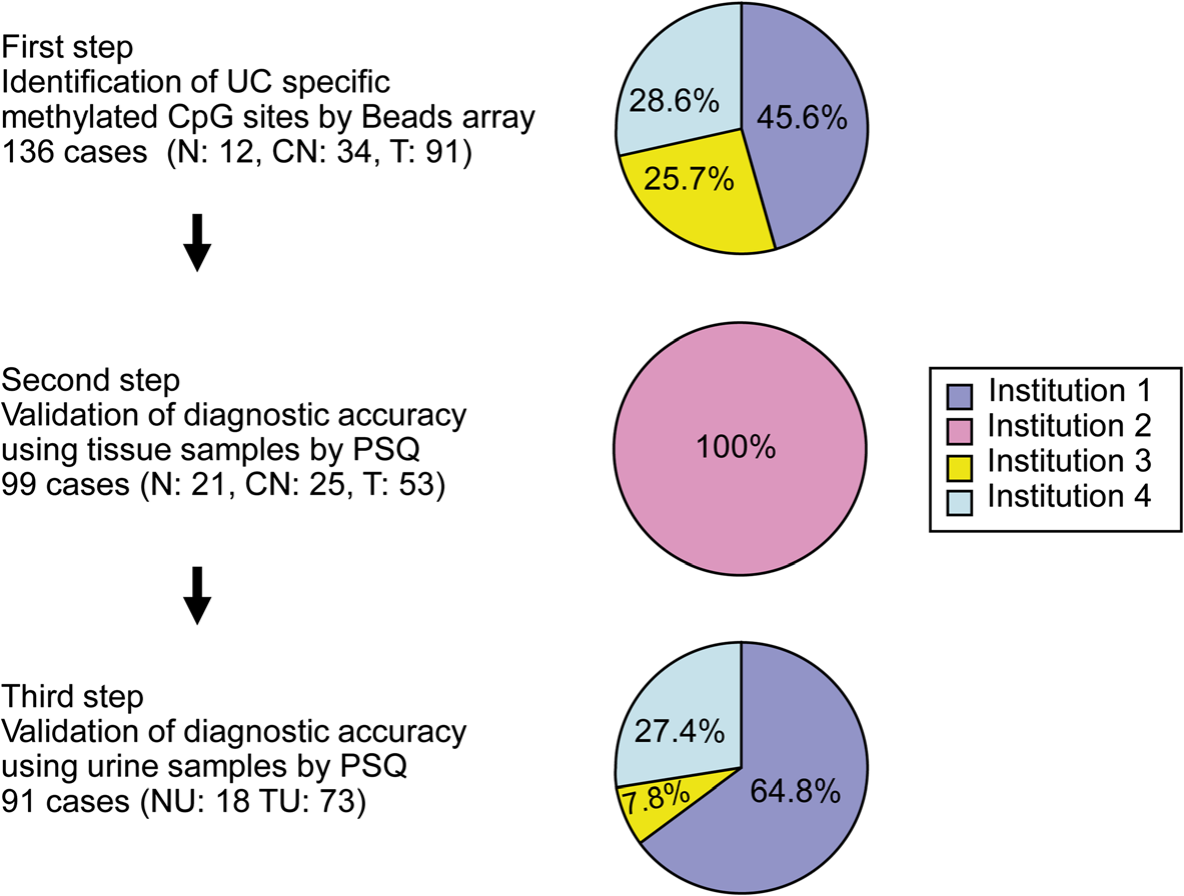
**Study design.** Samples of urothelial tissues and urine collected at the indicated participating centers and distributed for identification of UC-specific DNA-methylation sites (**First step**) and validation of diagnostic accuracy (**Second and Third steps**) as indicated. N: normal urothelia, CN: corresponding normal-appearing tissue adjacent to tumor from UC patient, T: tumor samples from UC patients; NU: urine from normal participants, TU: urine from UC patients treated by transuretheral resection; PSQ: pyrosequencing. Institution 1: Department of Urology, Norris Comprehensive Cancer Center, University of Southern California. Institution 2: Urology Division, National Cancer Center Hospital, Tokyo. Institution 3: Department of Urology, Nara Medical University. Institution 4: Department of Urology, Tochigi Cancer Center Hospital.

Samples of urothelial tissue from UC patients (n = 144), adjacent normal appearing urothelia (n = 59) and patients without UC (n = 33) were divided into different experimental groups in order to generate sets for training and validation (Table 1). Samples of urine sediments from UC patients (n = 73) and healthy volunteers (n = 18) were analyzed as an independent validation sets. Samples collected from the 4 participating centers were distributed for identification of UC-specific DNA methylation and then for validation (Figure 1).

**Table 1:** Clinical characteristics of UC and control patients

	**Training set**	**Tissue validation set**	**Urine validation set**
**Control patients** (**n** = **51**)	**12** (**N**)	**21** (**N**)	**18** (**NU**)
Age, median (range) (years)	63 (50–80)	62 (27–82)	54 (16–77)
Male/female	12/0	13/8	6/12
**UC patients** (**n** = **217**)	**91** (**T**)	**53** (**T**)	**73** (**TU**)
Age, median (range) (years)	66 (40–91)	69 (49–85)	69 (36–88)
Male/female	80/11	42/11	59/14
**Tumor**-adjacent normal tissue^*^	**34** (**CN**)	**25** (**CN**)	-
**Tumor Stage in UC patients**			
Ta	20	2	7
T1	32	16	30
T2	13	9	24
T3	20	21	10
T4	6	5	2
**Tumor Grade in UC patients**			
G1	5	0	5
G2	38	25	32
G3	48	28	36

*Samples of normal-appearing tissue adjacent to the tumor were collected from UC patients for each set. Abbreviations: *N* normal urothelial tissue, *CN* corresponding normal-appearing tissue adjacent to the tumor in UC patients, *T* tumor tissue, *NU* urine sediments from healthy volunteers, *TU* urine sediments from UC patients.

Urothelial tissue samples were collected during surgical procedures from UC and control patients. Urine samples were collected from UC patients and healthy volunteers. Samples were divided into experimental groups as given.

### DNA methylation profiling using universal beads™ array

In our previous study, DNA methylation profiling was performed using the GoldenGate Methylation Cancer Panel I (Illumina Inc., La Jolla, CA) at the USC Epigenome Center [17]. In this study, the data were reanalyzed with the same platform for selected CpG sites from regions of aberrant DNA methylation specifically associated with tumors. The array interrogated 1,505 CpG sites selected from 807 cancer-related genes. The data were first analyzed using the BeadStudio Methylation software (Illumina Inc., La Jolla, CA), and then a supervised cluster analysis with correlation metrics and average linkage was carried out using the open-source program Cluster 3.0. A β value of 0 to 1.0 was reported for each CpG site signifying percent methylation from 0-100%, respectively. The β values were calculated by subtracking background using negative control on the array and calculating the ratio of the methylated signal intensity to the sum of both methylated and unmethylated signals plus a constant of 100. Measurements with detection p > 0.05 were marked missing.

### Bisulfite pyrosequencing

DNA methylation status of candidate tumor-specific hyper- or hypo-methylated CpG sites was assessed by pyrosequencing (PSQ) using Pyrosequencing 96HS (Biotage, Uppsala, Sweden) and PyroMark Q24 (Qiagen) according to the manufacturer’s protocol. To enable single-strand preparation, the reverse primer was 5′-biotinylated. Reaction volumes of 30 μl contained 5× GoTaq buffer, 1.5 units GoTaq Hot Start Polymerase (Promega), 1 μM of primers, and 500 nM of dNTPs. PCR conditions were as follows: 95°C for 3 min; 45 cycles of 95°C for 30 s, the respective annealing temperature for 30 s, and 72°C for 30 s; and a final extension step at 72°C for 4 min. PCR primer sequences are given in Table 2. PSQ primers were designed to include CpG or near-CpG regions within 300 bps that were assayed on the Illumina GoldenGate Panel.

**Table 2:** Primer sequences for PSQ

**Gene**	**Annotation**	**Forward**	**Reverse**	**Sequencing**	**Sequence analyzed**	**Amplicon location relative to transcription start site**
*SOX1*	Sex determining region Y box1	GGTATTTGGGATTAGTATATGTTTAG	CTATCTCCTTCCTCCTAC	TTAGTATATGTTTAG	CGTACGCGGCGCGTCG	-462~ -351
*TJP2*	Tight junction protein 2	GGTTTTTAGATAGGATTTAAAATTTTGAG	CAAAACCTCACACAAACAACTTC	AGGTTTTTTTAGTT	CGATTTTTCG	-492~ -409
*MYOD1*	Myogenic differentiation 1	GTGGGTATTTAGATTGTTAGTA	ACAATAACTCCATATCCTAAC	GAAGTTAGGAT	CGTGTCGCGTTATCG	+96~ +233
*HOXA9_1*	Homeo box A9	TTGTTTAATTTTATGTGAGGGGTTT	CAAATCTAACCTTATCTCTATACTCTCCC	TGATATAAAATAGTT	CGTTTAAG	-397~ -243
*HOXA9_2*	Homeo box A9	ATGAAATTTGTAGTTTTATAATTTT	ATTACCCAAAACCCCAATAATAAC	GTTTTATAATTTT	CGTGGGTCGGGTCGGGCGG	+10~ +100
*GALR1*	Galanin receptor 1	ATTAATGGA TGAGGAGGTT	ATACCAAAAA CTTCTCTACT AC	GTGATTTTTA AGGGG	CGCGGATTTT AGTCGAGTTG	-194~ +110
*IPF1*	Insulin promoter factor 1	GTAGTTTTAA GAGGAAGG	AAAAATTAAA ACCCATTTAA CCAA	GTAGTTTTAA GAGGAAGGT	CGCGTTTTTTTTTTTCGTTG	-786~ -702
*TAL1*	T-cell acute lymphocytic leukemia 1	GTAAATAGAA GGAGGTTTT	ACACTACTTT CAAAAATATA AC	AGAA GGAGGTTTT	CGTAG TTAATTTAAG ATTTCG	-613~ -470
*EYA4*	Eyes absent homolog 4	GGATGTTTTGTTTTTATTAGAGGTATAG	AATTCTCTCAACTCAAACTCCC	GAAGGGGAAATTT	CGATATTGGAAGGAACG	+252~ +457
*CDH13*	Cadherin 13	AGTTTAAAGAAGTAAATGGGATGTTA	CTTCCCAAATAAATCAACAACAAC	ATTTGTTATGTAAAA	CGAGGGAGCGT	-175~ +6
*CYP1B*	Cytochrome P450 family 1	GTTTTGATTTTGGAGTGGGAGT	CTACCCTTAAAAACCTAACAAAATC	AGGGTATGGGAATTGA	CGTTATTTATCGA	+26~ +178
*NPY*	Neuropeptide Y	GGGTTGTTTT TATTTTTGGT AGGATTAGA	CACCAAAACC CAAATATCTA CCC	AGGAAAGTAGGGAT	CGGGT ATTGTTCGAG	-353~ -253
*VAMP8*	Vesicle-associated membrane protein 8	AAGTTTTTGT TTGGGAAGTT ATT	CATATCTCAA AACAACCCAA	GTTAGGTGTG GTTGGAG	CGATTCGAGATGCGAGGTGG	-157~ +56
*CASP8*	Caspase 8	GAAGTTTGATTTTGTTGGTTTAAAA	CAACCTCTCTAACTAAACCCTCCTT	TGTTTAGAGGTTG	CGGGTTGCGGGT	+431~ +533
*SPP1*	Secreted phosphoprotein 1	GGAATAAGGA TAGGTAGGT	CAAAATAACT ACTTAAAAAA ACTACTTCAA	GAATAAGGAT AGGTAGGTTG GG	CGATTTGTTTAAGGTTGTAT	+99~ +117
*CAPG*	Capping protein	GGGGTAGGTTGGAAGGAAGA	ACAACCACCCTACCACCTTCA	GTTGGAAGGAAGA	CGAATTTACGAAGT	+200~+294
*RIPK3*	Receptor-interacting serine-threonine kinase 3	GTTTTTGGAA GGTGAGGAT	AAAACTAATA CCTTTCTCCT TAACT	ATTTAATT TGGTTG	CGGT AGGTGTTTAG GAAACG	-137~ -27
*IFNG*	Interferon gamma receptor 1	AATAGTATTTGTTTGTGGTTGAA	TAACACCAAATCTCAAAATAACT	GAAAATGATTGAATAT	CGATTTG	+257~ +359
*HLADPA1*	Major histocompatibility complex, class II, DP alpha 1	AATTTTGAAAATGAATTGTGAATTG	CATTCTCTATTACTAAATAAAAAAAAC	GAGTTTTTTTGATTA	CGTTGGTA	-74~ +38

### Immunohistochemistry

The immunohistological studies of *SOX1*, *TJP2*, *VAMP8* and *SPP1* were carried out on formalin fixed, paraffin embedded tissue samples, of which 5 normal tissues and 53 tumor tissues in the training set as described previously [19]. The primary antibodies were polyclonal rabbit anti-SOX1 (Abcam Inc., diluted at 1:500), polyclonal rabbit anti-TJP2 (kindly provided by Dr. Masuo Kondo, Graduate School of Pharmaceutical Sciences, Osaka University, Japan), monoclonal rabbit anti-VAMP8 (Abcam Inc., diluted at 1:100) and monoclonal rabbit anti-SPP1 (Abcam Inc., diluted at 1:100). Immunoreactivity was evaluated according to modified Allered’s score system [20]. Briefly, the score represented the estimated proportion of positively stained cells (0 = none, 1 = less than 1/100, 2 = 1/100 to less than 1/10, 3 = 1/10 to less than 1/3, 4 = 1/3 to less than 2/3, and 5 = 2/3 or above). The staining intensities were averaged from the positive cells (0 = none, 1 = weak, 2 = intermediate, and 3 = strong). The product of these scores served as the total score. All results were scored by one of the authors (H. K.) without prior knowledge of the DNA methylation status.

### Statistical analysis

Graphpad Prism version 4.02 was used for performing the Mann–Whitney *U* test, calculating receiver operating characteristics (ROC) for sensitivity and specificity of the candidate loci and Pearson’s correlation coefficient.

## Results

### Identification of candidate UC-specific aberrant DNA-methylated CpG Sites

**Figure 2:**
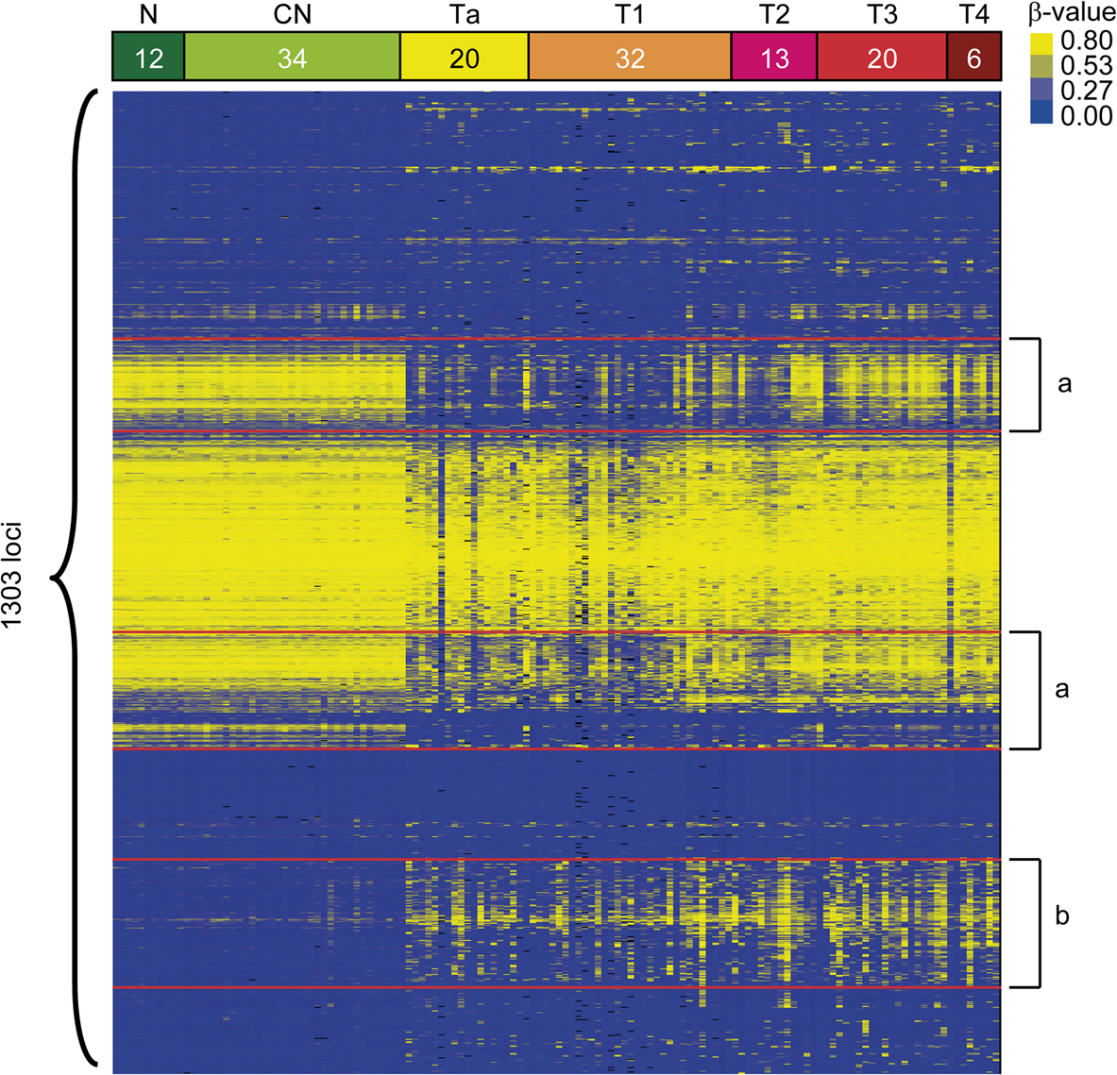
**Global DNA methylation alterations in UC.** Supervised cluster analysis of 1,303 loci (784 genes) from bladder samples, using the Illumina GoldenGate methylation assay. N (n = 12) represents normal tissue from patients without urothelial cancer (UC); CN (n = 34) represents corresponding normal-appearing tissue from UC patients; Ta-T1 (n = 49) represents non-muscle-invasive bladder cancer; and T2-T4 (n = 38) represents muscle-invasive bladder cancer. No methylation is shown in blue, and increasing DNA methylation is shown in yellow. (**a**) UC-specific hypomethylated CpG sites, and (**b**) UC-specific hypermethylated CpG sites.

In our previous study, differentially methylated regions had been identified in DNA samples from normal and UC urothelial tissues [17]. In the present study, as a first step, tumor-specific, aberrant DNA methylation sites were identified within CpG loci. DNA methylation profiling was compared between 3 groups of tissue samples (Figure 2): normal urothelial tissue (N, n = 12), corresponding normal-appearing tissue adjacent to the tumor in UC patients (CN, n = 34), and tumor samples saved during TUR procedure on UC patients (T, n = 91). The tumor samples were further stratified based on tumor staging into NMIBC and MIBC (Figure 2). X-linked CpGs and those with a poor signal (defined by a detection p-value of >0.05) were eliminated, which left 1,303 sites for analysis (Additional file 1: Table S1). A supervised cluster analysis of N versus CN and T samples revealed UC-specific DNA methylation alterations, of which 158 were hypermethylated CpG sites and 356 were hypomethylated sites (p < 0.001) (Figure 2, Additional file 2: Table S2). In these loci, we selected top 30 CpG sites from the statistical results which showed lesser p-value both between N and CN, also CN and T. We verified DNA methylation status using the same training sets by PSQ and compared with GoldenGate data. Finally, we identified the 12 CpG sites (5 were hyper methylated and 7 were hypomethylated) from 11 genes, of which quantification of DNA methylation status were well accorded with GoldenGate data (Table 3). We also identified the top 13 CpG sites which distinguished N from CN. Then PSQ was performed on DNA samples allocated to the tissue validation set (Table 1: 21Ns, 25 CNs and 53 Ts) and urine validation sets (Table 1: 18 urine sediments from healthy volunteers (NUs) and 73 urine sediments from UC patients (TUs)).

**Table 3:** ROC analysis of DNA methylation markers for UC

**Gene**	**Cut**-**off value (%)**	**AUC**	**Sensitivity (%)**	**Specificity (%)**	**P value**
**Validation in tissue (N/CN vs. T)**					
Hypermethylation					
*SOX1*	32.59	0.97	93.62	97.5	5.13E-14
*TJP2*	71.42	0.92	84.91	97.78	1.19E-12
*MYOD*	26.0	0.91	75.0	79.83	1.73E-12
*HOXA9*_*1*	55.59	0.86	76.6	97.83	9.00E-08
*HOXA9*_*2*	29.06	0.86	83.02	97.83	5.22E-10
Hypomethylation					
*VAMP8*	12.5	0.96	94.34	97.83	2.22E-15
*CASP8*	23.18	0.96	94.34	95.65	4.88E-15
*SPP1*	26.14	0.95	86.79	100	1.49E-14
*IFNG*	64.7	0.93	82.98	95.65	2.16E-12
*CAPG*	16.21	0.93	83.02	95.65	1.08E-12
*HLADPA1*	14.31	0.88	84.62	86.96	1.06E-09
*RIPK3*	22.97	0.85	81.63	84.44	9.54E-07
**Validation in tissue (N vs. CN)**					
Hypermethylation					
*SOX1*	16.51	0.86	68.18	100	9.04E-05
*MYOD*	12.71	0.85	76.0	85.71	5.19E-05
*HOXA9_1*	22.95	0.80	76.0	80.95	0.00043
*GALR1*	7.24	0.85	76.0	85.71	4.26E-05
*IPF1*	33.83	0.74	64.0	76.19	0.0089
*TAL1*	29.47	0.83	76.0	85.71	0.00011
*EYA4*	6.38	0.80	83.33	73.68	0.0078
*CDH13*	7.13	0.93	88.0	85.71	5.24E-07
*CYP1B*	13.61	0.75	60.0	80.95	0.0040
*NPY*	10.31	0.82	88.0	71.43	0.00018
Hypometylation					
*CASP8*	46.38	0.73	60.0	85.71	0.0084
*IFNG*	84.93	0.78	56.0	95.24	0.001
*HLADPA1*	24.27	0.83	72.0	85.71	0.00011
**Validation in urine sediment (NU vs. TU)**					
Hypermethylation					
*SOX1*	15.62	0.74	41.54	100	0.0041
*TJP2*	7.933	0.79	92.54	56.25	0.0003
*MYOD*	9.897	0.93	86.79	87.50	3.10E-05
*HOXA9_1*	7.038	0.92	86.23	88.89	4.25E-05
*HOXA9_2*	3.20	0.81	88.57	61.54	0.0004
Hypomethylation					10.78
*VAMP8*	10.78	0.72	97.06	40.0	0.023
*CASP8*	7.863	0.82	73.61	76.92	0.0005
*SPP1*	21.23	0.79	85.94	75.0	0.0015
*IFNG*	86.08	0.76	55.07	92.31	0.0037
*CAPG*	8.08	0.67	83.82	56.25	0.04
*HLADPA1*	6.46	0.82	77.19	90.0	0.0009
*RIPK3*	9.37	0.75	82.35	70.0	0.011

Selected loci that were identified as either hyper- or hypo-methylated were analyzed for their degree of DNA methylation and association with UC. The loci are named by the genes in which they occur; if there are 2 loci in the same gene, the suffixes 1 and 2 are added.

### Diagnostic accuracy of DNA methylation markers of UC

In the next step, the sequence-verified loci were tested for diagnostic accuracy by ROC analysis. To determine the diagnostic accuracy for UC tumors, T versus N/CN analysis was performed on 12 CpG loci from 11 genes, of which 5 loci were hypermethylated and 7 hypomethylated (Table 3). The cut-off values to discriminate T from N/CN using each marker were determined from the ROC curves as the maximum values of sensitivity and specificity, as follows: [sensitivity (%) + specificity (%) – 100]. For all 12 loci, there was a statistically significant and dramatic distinction in DNA methylation levels between N/CN and T. The ranges for area under the curve (AUC), sensitivity and specificity were 0.85–0.97, 75.0–94.34% and 84.44–100% respectively (Table 3). In particular, combination analysis of *SOX1* and *VAMP8* could distinguish T from N/CN with 100% sensitivity and specificity (data not shown). Interestingly, DNA methylation levels in CN samples were not correlated with their respective T samples, and DNA methylation levels in T samples did not correlate with age, gender and stage for all 12 markers.

To determine the diagnostic accuracy of epigenetic field defect, ROC analysis was performed for the tissue samples, N versus CN, using 13 markers from 13 genes, of which 10 were hypermethylated and 3 hypomethylated (Table 3). The ranges for AUC, sensitivity and specificity were 0.73–0.93, 56.0–88.0%, and 71.43–100%, respectively (Table 3).

Diagnostic accuracy for UC as measured by DNA methylation in urine samples was evaluated based on the same 12 loci as for tissue samples, and determined by ROC analysis on NU versus TU urine samples. For all 12 markers, DNA methylation levels in TUs were statistically significantly distinct from those in CUs. The ranges of AUC, sensitivity and specificity were 0.67–0.93, 41.54–97.06%, and 40.0–100% respectively (Table 3). Among the loci examined here, values for AUC corresponding to urine samples were lower than those corresponding to urothelial tissues, except for the loci *MYOD* and *HOXA9*_*1*. Also the cut-off value which distinguishes TU from NU in both hyper- and hypo- methylated markers were lower in urine than in the tissue for all cancer types, except in *IFNG*. These results suggested that either the copy number of methylated CpG loci in urine sediments was difficult to be detected because of low DNA quality, or the concentration of cancer cells were diluted by the presence of other unrelated cells in the urine.

**Figure 3:**
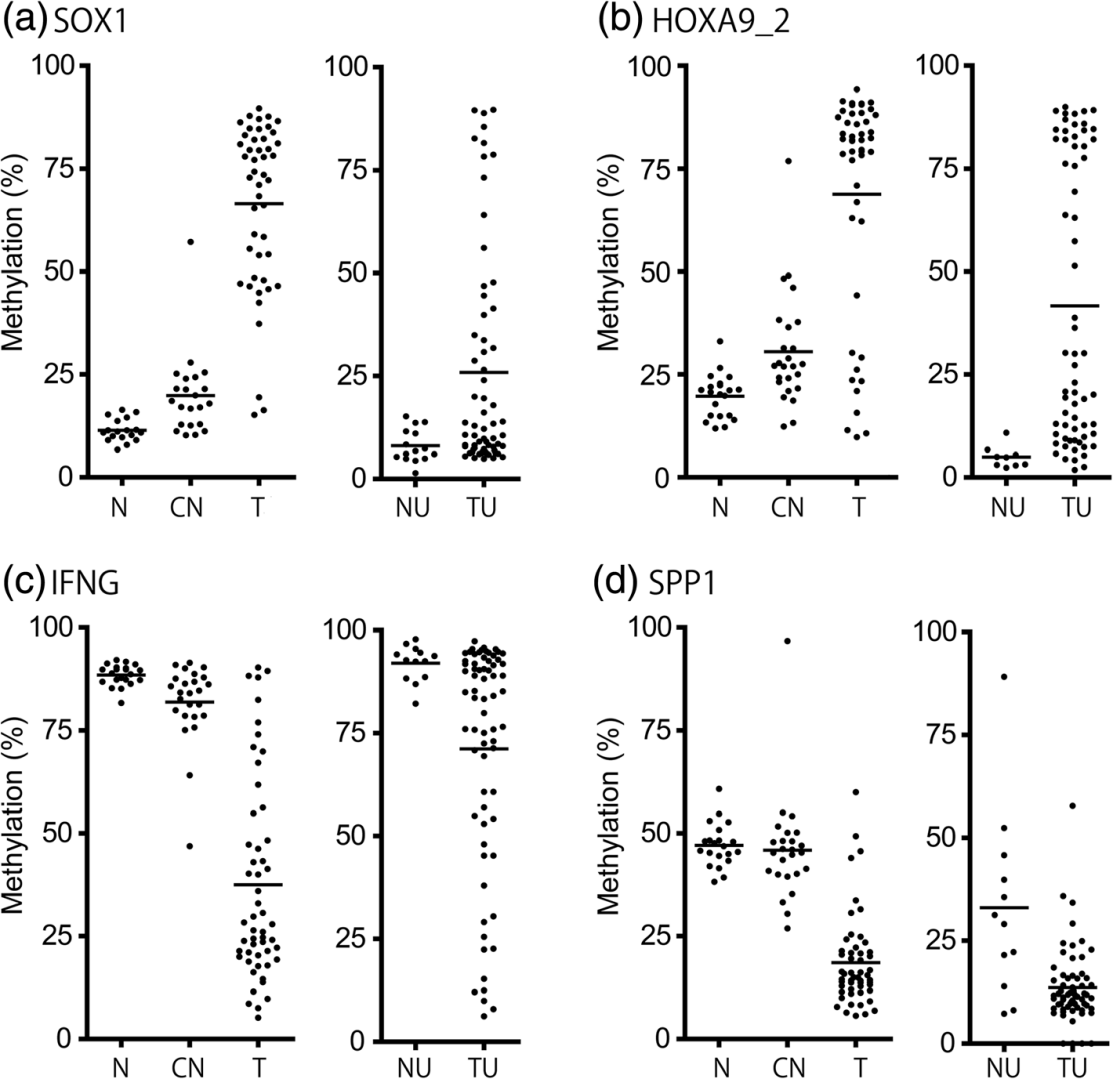
**Differential DNA methylation at CpG sites.** Scatter plots of quantitative DNA methylation analysis by PSQ in select loci that were hypermethylated: (**a**) *SOX1* (**b**) *HOXA9_*x*2*; or hypomethylated: (**c**) *IFNG* (**d**) *SPP1*. Mann–Whitney *U* test was used to compare quantitative methylation levels between the 2 groups. Short horizontal lines represent the median.

Representative scatter plots for 2 hypermethylated loci (*SOX1* and *HOXA9*_*2*) and 2 hypomethlated loci (*IFNG* and *SPP1*) examined in the various tissue and urine samples are shown (Figure 3).

The DNA methylation data were analyzed for each tissue/urine sample to determine the number of loci for which a given sample was considered a true positive based on the respective cut-off value (Table 4). Thus, out of the 53 T samples, 50 were positive for at least 6 and more loci. On the other hand, there were 3 T samples that were false negative for some loci and there was 1 N/CN sample that was false positive for some loci. Most tumor samples were positive for at least 6 markers. In other words, true-positive levels of DNA methylation for 6 or more markers allowed clear discrimination between T and N/CN samples with 94.3% sensitivity and 97.8% specificity (Table 4 top). For distinguishing between cancerous and non-cancerous tissue, the 13 loci selected for comparing N (n = 21) with CN samples (n = 25) were examined for each tissue sample. All the normal samples were positive for a maximum of 6 loci, while a majority of the CN samples were positive for at least 8 loci. Hence, for samples that showed altered DNA methylation for 7 or more markers, N could be discriminated from CN with 76.0% sensitivity and 100% specificity (Table 4 middle; false negative: 6/25; false positive: 0/21). In the case of urine samples, the 12 loci with altered DNA methylation were examined for each sample of the NU (n = 18) and TU (n = 73) groups (Table 4 bottom). The distinction between the 2 groups was clear as there were no false positives or false negatives and all TU samples were positive for at least 6 loci. Thus, in the case of samples that showed true-positive levels of altered DNA methylation in 6 or more loci, discrimination between TU and NU samples was possible with 100% sensitivity and 100% specificity.

**Table 4:** Diagnostic accuracy of the panel markers for UC

				
	Aberrant methylation		Sensitivity (%)	Specificity (%)
	Less than 5	6 and more		
N/CN	45	1	94.3	97.8
T	3	50		
	Aberrant methylation		Sensitivity (%)	Specificity (%)
	Less than 6	7 and more		
N	21	0	76	100
CN	6	19		
	Aberrant methylation		Sensitivity (%)	Specificity (%)
	Less than 5	6 and more		
NU	18	0	100	100
TU	0	73		

Abbreviations: *N* normal urothelial tissue, *CN* corresponding normal-appearing tissue adjacent to the tumor in UC patients, *T* tumor tissue, *NU* urine sediments from healthy volunteers. *TU* urine sediments from UC patients.

### Correlation of the genetic expression with DNA methylation status

To evaluate epigenetic gene regulation of UC-specific aberrant DNA-methlated CpG sites, we made a comparison between DNA methylation levels and genetic expression on 2 hypermethlated and 2 hypomethylated markers. In hypermetylated genes, *SOX1* expression decreased in tumor tissues significantly (p = 0.0107). However DNA methylation levels did not correlate with gene expression (Additional file 3: Figure S1). On the other hand, gene expression of 2 hypomethlated genes significantly increased in tumor tissues. Furthermore DNA methylation levels of *SPP1* inversely correlated with gene expression significantly.

## Discussion

Earlier studies have shown distinct DNA methylation patterns between UC and normal tissues, which could serve as useful indicators of early stages in the multi-step process of carcinogenesis in UC [9,10]. Further, urothelial tissues affected by UC could be clearly distinguished from normal urothelia based on the presence of aberrant DNA methylation regions in cancer-associated genes such as *CDH1*[21], *RASSF1A*[11] and *RUNX3*[22] with sufficient sensitivity and specificity. However, to diagnose UC via analysis of a urine sample, a combination of several DNA methylation markers would be required to ensure high accuracy. Hence, the aberrant DNA methylation status of previously reported UC-associated genes alone would not provide sufficient accuracy with high sensitivity and specificity. On the other hand, increasing the number of markers increases the sensitivity, albeit at the cost of specificity.

In this study, we identified a panel of loci with UC-specific alterations in DNA methylation. The study design included 3 steps for identification and validation of these loci analyzed in urothelial tissue or urine samples (Figure 1). In the first step, high-throughput DNA methylation profiling revealed a total of 514 CpG sites that caused UC-specific aberrant methylation with statistical significance (p < 0.001). This corresponds to 39.4% of CpG sites assayed by the Bead™ array and suggested genome-wide UC-specific DNA methylation. Furthermore, normal tissue and normal-appearing tissue adjacent to UC patients were found to be significantly different with regard to 39 hypermethylated sites and 7 hypomethylated sites. These CpG sites could also be used to diagnose UC risk. (data not shown). These results indicated that aberrant DNA methylation in UC already occurred in non-cancerous epithelia in UC patients, supporting the notion that DNA methylation alterations occur gradually during the multistep process of carcinogenesis.

The DNA methylation status of the various CpG sites identified from Bead™ array data as UC-specific was sequence verified by PSQ. Next, we evaluated the diagnostic accuracy of 12 CpG sites. Interestingly, most of these loci were in genes that have not been reported for their aberrant DNA methylation in UC, except *CASP8*[23]. Since these CpG sites were identified from the clustering data in the comparison of normal and cancerous tissues, DNA methylation levels assayed by PSQ represented the fraction of methylated DNA clones in a sample, proportional to the number of malignant cells, if the tumor heterogeneities are ignored. In the tissue analysis, DNA methylation level between N/CN and T could be clearly discriminated for each marker, and the combination analysis of all 12 markers provided accuracy, 94.3% sensitivity, and 97.8% specificity (Table 4). Furthermore, CN could be discriminated from N with 76.0% sensitivity and 100% specificity. These results indicate that UC-specific aberrant DNA methylation also occurred in the adjacent normal epithelia, but at a lower level than in the tumor. In this way, the quantitative methylation analysis has an advantage in detecting field defect, which is a useful indicator for determining UC risk or predicting recurrence. Aberrant DNA methylation of *TJP2*, *SPP1*, and *IFNG* did not show a statistically significant difference between N and CN (data not shown), although these epigenetic alterations are thought to be cancer-specific and a part of the multistep carcinogenesis. Interestingly, *TJP2* (tight junction protein) is located on chromosome 9 (9q21.11), which shows allelic loss in UC most frequently. Allelic loss on chromosome 9 was thought to be the earliest genetic event arising in UC; however, we previously reported that allelic loss on 9q had not occurred in tissue showing dysplasia and adjacent normal urothelia of UC patients [19]. Taking into consideration these genetic and epigenetic alterations in adjacent normal urothelia, the alteration on 9q might be a truly tumor-specific event.

In the urine analysis, the combination of 12 markers provided sufficient accuracy to discriminate TU from NU, with 100% sensitivity and 100% specificity, and indicated a higher detection value for UC than so far reported for DNA methylation marker panels using quantitative analysis [13,14]. However, compared with the tissue analysis, the diagnostic power of each marker was not sufficient, and data from all 12 markers were required for a true diagnosis.

To determine whether the aberrantly methylated loci might play a functional role in tumorigenesis, we compared 4 genes expression to DNA methylation levels. In our results, a hypermethylated gene, *SOX1* expression reduced in tumor tissue, whereas *TJP2* expression did not reduce. In a recent study by Dudziec E. et al. [24], a large scale profiling among DNA methylation, histone modification and gene expression using UC cells revealed that 20-30% genes were silenced by epigenetic regulation. In this way, aberrant regional hypermethylation in cancer cells do not always regulate gene expression, and the hypermethylated loci that identified in this study might be a hallmark of cancer. In contrast to promoter hypermethylation, hypomethylation-dependent transcriptional activation in cancer is less frequent [25]. Currently, major contribution of global hypomethylation especially in retrotransposons and pericentromeric repeats are thought to be the enhancement of genomic instability [26]. Interestingly, hypomethylation of *VAMP8* and *SPP1* correlated with the gene expression significantly. Furthermore DNA methylation levels of *SPP1* inversely associated with expression levels. Several studies showed some transcription control regions, with the hypormethylated and activated in cancer [27,28] (Although we examined only 4 genes, our results might support these phenomena. Further studies needs to clarify the association aberrant DNA methylation with gene expression in cancer.

A limitation of this study is that candidate UC-specific DNA methylation loci were identified using tissue samples in the first step, and these markers showed a poorer diagnostic sensitivity in urine than in tissue samples. However, urine sediments from the healthy population sometimes show aberrant DNA methylation that is unrelated to cancer, and cluster analysis to identify DNA methylation loci by just urine samples may reflect the etiology of UCs. Another limitation is small numbers of each step. Also the consecutive concordant study that revealed DNA methylation status of T, CN and TU samples in one person including follow-up urines.

## Conclusions

In conclusion, by a genome-wide analysis, markers based on DNA methylation were identified for high accuracy of diagnosis of UCs using urine samples in our preliminary study. These markers will need to be validated in a larger scale study. In the future, it may be possible to develop a panel of carefully selected DNA methylation markers for use on urine sediments to detect both primary UCs and recurrent UCs. In this way, DNA methylation profiling might be a useful tool to discriminate several clnicopathological factor of UCs and to clarify the multi-step carcinogenesis of UCs.

## Competing interests

The authors declare that they have no competing interests.

## Authors’ contributions

YC conceived of the study, participated in its design and coordination and drafted the manuscript. YK and HF collected UC samples and gain ethics committee approval to enroll this study at National Cancer Center Hospital Tokyo Japan. YK also helped to performed PSQ experiments. KS and KK collected UC samples and gain ethics committee approval to enroll this study at Tochigi Cancer Center Hospital, Utsunomiya Japan. GL and PAJ participated in the design, helped to perform statisitical analysis and collected UC samples and gain ethics committee approval to enroll this study at USC, LA, USA. KF and YH collected UC and healthy urine samples, and gain ethics committee approval to enroll this study at Nara medical university, Kashihara, Japan. HK participated in writing of the manuscript. All authors read and approved the final manuscript.

## Pre-publication history

The pre-publication history for this paper can be accessed here:

http://www.biomedcentral.com/1471-2407/13/275/prepub

## Supplementary Material

Additional file 1: Table S1 All data of universal beads™ array. Click here for file

Additional file 2: Table S2 Aberrant DNA methylated loci obtained from beads™ array. Click here for file

Additional file 3: Figure S3 Correlation between gene expression and DNA methylation levels in normal and UC tissues.Five normal urothelial tissues (N) and 53 tumor tissues (T) (Stage, Ta: 13, T1: 21, T2: 7, T3: 10, T4: 2, Grade, G1: 2, G2: 25, G3: 26) were analyzed. Immunohistocheistry (IHC)(left) represents corresponding median IHC score in each group. Original magnification, ×200. Expression of 4 genes in normal and tumor tissues were shown in Scatter plots (middle). Mann–Whitney *U* test was used to compare quantitative methylation levels between the 2 groups. Short horizontal lines represent the median. Pearson’s correlation coefficient between IHC score and DNA methylation levels (right). Blue circles represent normal tissues. Click here for file
