# Supplementary figures and images for: Diagnostic markers of urothelial cancer based on DNA methylation analysis
Source: BMC Cancer. 2013 Jun 4;13:275. doi: 10.1186/1471-2407-13-275 (PMC3691617; doi:10.1186/1471-2407-13-275)

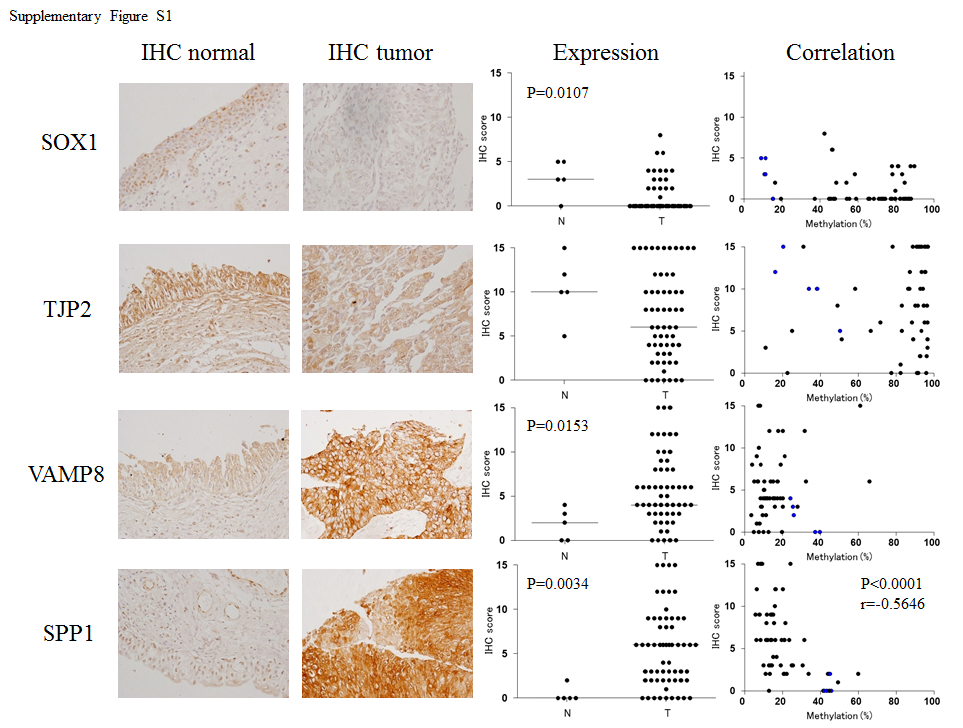

Supplement: Additional file 3: Figure S3 — Correlation between gene expression and DNA methylation levels in normal and UC tissues.Five normal urothelial tissues (N) and 53 tumor tissues (T) (Stage, Ta: 13, T1: 21, T2: 7, T3: 10, T4: 2, Grade, G1: 2, G2: 25, G3: 26) were analyzed. Immunohistocheistry (IHC)(left) represents corresponding median IHC score in each group. Original magnification, ×200. Expression of 4 genes in normal and tumor tissues were shown in Scatter plots (middle). Mann–Whitney U test was used to compare quantitative methylation levels between the 2 groups. Short horizontal lines represent the median. Pearson’s correlation coefficient between IHC score and DNA methylation levels (right). Blue circles represent normal tissues. [file 1471-2407-13-275-S3.tiff]
